# Socio-economic inequalities in mental health: a new framework and analysis across 113 countries

**DOI:** 10.1093/eurpub/ckac129.156

**Published:** 2022-10-25

**Authors:** C Henking

**Affiliations:** Department of Social Policy and Intervention, University of Oxford, Oxford, UK

## Abstract

**Background:**

Socio-economic inequalities in common mental health disorders (CMDs) cut across each step in the cascade of care: (1) Less affluent individuals have a higher prevalence of CMDs, (2) are less likely to utilise treatment and (3) might benefit less from treatment when they do receive it. Here, we propose a new framework for distinguishing between these three types of inequalities in CMDs and test if such ‘triple inequalities’ exist globally and how they vary across countries.

**Methods:**

We use the Wellcome Global Monitor 2020 (N = 119,088 in 113 countries) to test if socio-economic factors, psychological factors (stigma and trust) and country-level factors (GDP, GINI and health expenditure) predict CMD lifetime prevalence, utilisation and perceived helpfulness of talking therapy and medication. Multi-level logistic regression models were used.

**Results:**

As predicted, people with higher household income are less likely to experience anxiety or depression (OR = 0.90 for each income quintile, p < 0.01), more likely to talk to a mental health professional (OR = 1.05; OR = 1.34 for higher education, p < 0.01) and more likely rate this treatment as very helpful (OR = 1.06, p = 0.02) across countries. In contrast, income is not linked with utilisation (OR = 0.99, p = 0.18) and helpfulness of ‘taking medication’ for CMDs (OR = 1.02, p = 0.26). In LMICs, the highly educated take less medication (OR = 0.74, p < 0.01). Local stigma reduces utilisation (OR = 0.95) and helpfulness of talking therapy (OR = 0.77), while trust in health practitioners increases both (OR = 1.07 util. and OR = 1.31 helpf., p < 0.01 in all cases).

**Conclusions:**

Three types of socio-economic inequalities for CMDs (in prevalence, talking therapy utilisation and helpfulness) deepen disadvantages for the less affluent across 113 countries. For pharmacological treatment, inequalities in utilisation and helpfulness are weaker and have a different social gradient in LMICs. Here, less educated people are more likely to take medication.

**Key messages:**

• Three types of socio-economic inequalities in common mental health disorders (in prevalence, talking therapy utilisation and helpfulness) exacerbate disadvantages for less affluent individuals.

• These Inequalities in CMD treatment utilisation and helpfulness are stronger for talking therapies than for medication, and depend on country contexts, stigma and trust in health practitioners.

